# Real-world patterns of post-progression treatment and outcomes in patients with HR+/HER2− advanced breast cancer treated with CDK4/6 inhibitors

**DOI:** 10.1093/oncolo/oyag003

**Published:** 2026-01-11

**Authors:** Rosalba Torrisi, Federica Giugliano, Laura Giordano, Giuseppe Saltalamacchia, Flavia Jacobs, Ambra Carnevale Schianca, Monica Milano, Nadia Bianco, Claudia Anna Sangalli, Rita De Sanctis, Giovanna Masci, Giuseppe Curigliano, Armando Santoro, Elisabetta Munzone

**Affiliations:** Medical Oncology and Hematology Unit, Humanitas Research Hospital IRCCS, Rozzano, MI 20089, Italy; Division of New Drugs and Early Drug Development for Innovative Therapies, European Institute of Oncology, IRCCS, 20141 Milan, Italy; Department of Oncology and Hemato-Oncology, University of Milan, Milan 20122, Italy; Medical Oncology and Hematology Unit, Humanitas Research Hospital IRCCS, Rozzano, MI 20089, Italy; Medical Oncology and Hematology Unit, Humanitas Research Hospital IRCCS, Rozzano, MI 20089, Italy; Medical Oncology and Hematology Unit, Humanitas Research Hospital IRCCS, Rozzano, MI 20089, Italy; Division of New Drugs and Early Drug Development for Innovative Therapies, European Institute of Oncology, IRCCS, 20141 Milan, Italy; Department of Oncology and Hemato-Oncology, University of Milan, Milan 20122, Italy; Division of Medical Senology, European Institute of Oncology, IRCCS, Milan 20141, Italy; Division of Medical Senology, European Institute of Oncology, IRCCS, Milan 20141, Italy; Scientific Direction, European Institute of Oncology, IRCCS, Milan 20141, Italy; Medical Oncology and Hematology Unit, Humanitas Research Hospital IRCCS, Rozzano, MI 20089, Italy; Department of Biomedical Sciences, Humanitas University, Pieve Emanuele, MI 20072, Italy; Medical Oncology and Hematology Unit, Humanitas Research Hospital IRCCS, Rozzano, MI 20089, Italy; Division of New Drugs and Early Drug Development for Innovative Therapies, European Institute of Oncology, IRCCS, 20141 Milan, Italy; Department of Oncology and Hemato-Oncology, University of Milan, Milan 20122, Italy; Medical Oncology and Hematology Unit, Humanitas Research Hospital IRCCS, Rozzano, MI 20089, Italy; Department of Biomedical Sciences, Humanitas University, Pieve Emanuele, MI 20072, Italy; Division of Medical Senology, European Institute of Oncology, IRCCS, Milan 20141, Italy

**Keywords:** HR+/HER2 negative advanced breast cancer, CDK4/6 inhibitors, post-CDK4/6 inhibitors treatment, post-progression outcomes

## Abstract

**Patients and methods:**

we retrospectively collected data of patients with HR+/HER2− advanced breast cancer (ABC) treated with endocrine therapy (ET) and a CDK4/6 inhibitor (CDK4/6i) aiming to describe the patterns of post-progression outcomes.

**Results:**

Among 452 evaluable patients 325 were treated in the first-line setting. Median progression-free survival (mPFS) was 22.8 months overall and 29.7 months in patients treated in first-line setting. Factors associated with outcomes in multivariate analysis were the line of CDK4/6i therapy, *de novo* vs recurrent disease, visceral vs bone-only metastases, and primary endocrine resistance.

A total of 300 patients progressed and 250 overall and 156 in the first-line cohort received a subsequent treatment. Visceral progression and CDK4/6i duration <12 months were associated with a higher likelihood of receiving anthracycline or taxanes (AT) as compared to ET ±everolimus (EET). Post-progression PFS (PPFS) and post-progression OS (PPOS) were statistically significantly better with EET and capecitabine (C) over AT overall and in patients with visceral progression. Multivariate analysis confirmed a significant advantage for EET and C, while visceral progression retained a significant impact only on PPOS. After progression to the first post-CDK4/6i treatment C obtained a significant better PPOS as compared to other treatments.

**Conclusion:**

We showed in a large real-world series that most patients with HR+/HER2− ABC failing CDK4/6i and ET unselected for the occurrence of molecular mutations retain endocrine sensitivity and may benefit of a subsequent ET ± a targeted therapy delaying the need for chemotherapy regardless of site of progression and prior CDK4/6i therapy duration.

## Graphical abstract

**Graphical Abstract:**
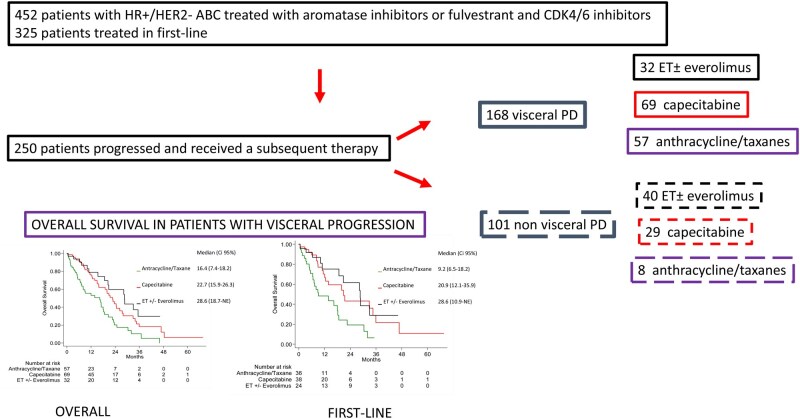

Implications for Practice No definite recommendations for treatment of HR+/HER2− advanced breast cancer after endocrine therapy (ET) plus CDK4/6 inhibitors (CDK4/6i) in absence of actionable mutations exist.

Our results show in a real-world series that most patients with HR+/HER2− ABC failing therapy with CDK4/6i and ET unselected for the occurrence of molecular mutations retain endocrine sensitivity and derive a meaningful clinical benefit from a subsequent endocrine manipulation ± everolimus delaying the need for chemotherapy regardless of site of progression and CDK4/6i therapy duration. These findings support a beneficial role for extending endocrine therapies in routine clinical practice when actionable mutations are not detected or not evaluable.

## Introduction

Approximately 80% of breast cancers are hormone receptor positive HER2 negative (HR+HER2−).^1^ The combination of endocrine therapy (ET) and CDK4/6 inhibitors (CDK4/6i) is the cornerstone of treatment for HR+/HER2− advanced breast cancer (ABC),^2^^,^^3^ having consistently doubled progression free survival (PFS) and prolonged overall survival (OS) in some settings.^4–9^

On the other hand, no definite recommendations on post-progression treatments exist since multiple mechanisms of resistance to CDK4/6i have been proposed and results with single treatments after CDK4/6i failure are lacking.^4–11^

Randomized clinical trials (RCTs) have shown that in patients with actionable molecular alterations further endocrine manipulations as oral Selective Estrogen Degraders (SERDs) like elacestrant and camizestrant for patients with *ESR1* mutations^12^^,^^13^ or *PI3KCA/AKT/mTOR* inhibitors as capivasertib, inavolisib, alpelisib for patients with mutations of that pathway are highly active.^14–17^

In the present retrospective study we collected patterns of progression and post-progression outcomes in terms of post-progression PFS (PPFS or PFS2) and post-progression OS (PPOS) in patients treated with CDK4/6i in routine clinical practice at two large-volume Institutions in order to describe treatment choice after progression and clinical features driving clinician’s choice and affecting outcomes.

## Patients and methods

A retrospective series of patients with HR+/HER2− ABC who received the combination of ET and CDK4/6i as part of their routine treatment at two Italian institutions from January 2016 to March 2023 was included (data cut off: September 30, 2024).

Eligible patients had received treatment with ET and CDK4/6i for at least 1-yr or had progressed before completing 1-yr of treatment; patients treated beyond the third-line were excluded in order to comply with current treatment guidelines and regulatory approvals of drugs.

All patients included in the study had advanced breast cancer at treatment initiation, defined as locally advanced disease not amenable to curative treatment and/or distant metastases. Approximately one-third of patients presented with *de novo* metastatic disease. Thereafter, any subsequent change in disease status was classified as progression, which was further categorized—for clinical purposes—as visceral or non-visceral progression.

Timelines of drug reimbursement by the Italian National Health System, evidence from randomized clinical trials available at the time of treatment start for specific features as menopausal and endocrine resistance status and expected tolerability according to comorbidities were the main determinants guiding the choice of CDK4/6i.

Patients were monitored at about 3-month intervals or less when progressive disease was suspected. CT scans, 18-FDG PET or MRI were used for monitoring disease according to the baseline modality used. Clinical examination was performed alternatively in case of skin involvement.

The objectives of the study were to describe in a real-world setting the patterns of progression and the post-progression outcomes (PPFS or PFS2 and PPOS) and whether clinical and pathological features as site of progression and duration of therapy with CDK4/6i affected treatment choice and outcomes.

Since this was a retrospective study response according to RECIST 1.1 criteria was not feasible in all patients, but CT scans and other imaging were reviewed in order to comply with RECIST 1.1 criteria whenever possible. Information on age at diagnosis, menopausal status at time of initiation of CDK4/6i, previous adjuvant therapies, number and response to previous therapies for metastatic disease, pathological features of primary and metastatic tumor when available, endocrine resistance, sites of metastatic disease at time of CDK 4/6i initiation, best response with ET+ CDK4/6i, site of disease progression if occurred, treatments and outcomes after progression and last follow-up visit were collected.

Endocrine resistance was defined according to the classical criteria^18^; we further distinguished patients who had never received ET as endocrine-naïve from endocrine-sensitive to investigate differences in outcomes according to some literature evidence which suggested better outcomes in patients diagnosed with *de novo* MBC reviewed in Torrisi et al.^19^

This study was approved by the institutional ethical committee of the coordinating center (Humanitas Research Hospital) and of the other participating Institution (European Institute of Oncology). All patients signed an informed consent. The study was conducted in compliance with Helsinki Declaration. The ESMO GROW criteria were followed in reporting the results of this study.^20^

## Statistical considerations

The study was planned as a retrospective evaluation of post-progression treatments of patients who had progressed on the combination of ET and CDK4/6i in a consecutive series of HR+/HER2− ABC patients.

Progression-free survival was defined as the time from the first day of treatment with ER and CDK4/6i to disease progression. Post Progression-Free Survival (PPFS or PFS2) was defined as the time from the first day of post-CDK 4/6i treatment until disease progression, as shown by radiological or clinical examination, or death from any cause. Patients without any evidence of progressive disease were censored at the date of their last follow-up. Post-progression curves were also analyzed by pattern of progression (visceral vs not visceral), type of therapy (ET or ET+ targeted therapy vs chemotherapy) CDK4/6i, and CDK4/6i treatment duration.

Progression after PFS2 (ie, after the first post-CDK 4/6i treatment) was defined as second progression or Post progression free survival 2(PPFS2). Patients without any evidence of progressive disease were censored at the date of their last follow-up. Post-progression curves were also analyzed by pattern of progression (visceral vs not visceral), type of therapy (ET or ET+ targeted therapy vs chemotherapy) CDK4/6i, and CDK4/6i treatment duration.

Survival curves were estimated using the Kaplan–Meier method. The impact of potential confounding factors, such as the pattern of progression (visceral vs. non-visceral), type of therapy (ET or ET + targeted therapy vs. chemotherapy), and the specific CDK4/6 inhibitor administered, was assessed. If a statistically significant effect was observed, these factors were included in the multivariable model. Differences between subgroups were analyzed using the log-rank test or the Cox proportional hazards model. Statistical significance was set at 0.050. Data were analyzed using SAS software version 9.4.

## Results

A total of 452 consecutive patients were evaluable for analysis. Table 1 summarizes baseline patient characteristics.

**Table 1.** Patient and tumor characteristics.

	Overall	First-line treated
	*N* (%)	*N* (%)
***N* patients**	452	325
**Age years mean (range)**	58 (20-91)	59 (20-91)
**MBC**		
** De novo**	154 (34.1)	115 (35.4)
** Recurrent**	298 (65.9)	210 (64.6)
**Menopausal status**		
** Pre-perimenopausal**	121 (26.8)	92 (28.3)
** Postmenopausal**	328 (72.6)	232 (71.4)
** Not applicable (male patients)**	3 (0.7)	1 (0.30)
**HER2 status**		
** HER2 0**	219 (48.5)	157 (48.3)
** HER2 low**	176 (38.9)	133 (40.9)
** Missing**	57 (12.6)	35 (10.7)
**Previous chemotherapy for MBC**	54 (12)	/
**Previous endocrine therapy**		
** Adjuvant**	275 (60.8)	194 (59.7%)
** MBC**	103 (22.8)	/
** Missing**		32 (9.9)
**Visceral disease**	239 (52.9)	155 (47.7%)
**Bone-only disease**	117 (25.9)	92 (28.3%)
**Biopsy**	363	267
** Breast**	90 (24.8%)	77 (25.1)
** Metastatic sites**	273 (75.2)	190 (71.2)
**Endocrine resistance**		
** Naïve**	131 (29)	123 (37.9)
** Hormone sensitive**	100 (22.1)	89 (27.4)
** Primary resistance**	53 (11.7)	38 (11.7)
** Secondary resistance**	151 (33.4)	74 (22.8)
** Missing**	17 (3.8)	1 (0.30)
**CDK 4/6i line of therapy**		
** 1st**	325 (71.9)	/
** 2nd**	85 (18.8)	/
** 3rd**	42 (9.3)	/
**CDK 4/6 i**		
** Abemaciclib**	77 (17)	69 (21.2)
** Palbociclib**	247 (54.7)	140 (43.1)
** Ribociclib**	128 (28.3)	116 (35.7)
**Endocrine agent**		
** Aromatase inhibitors**	240 (53.1)	220 (67.7)
** Fulvestrant**	212 (46.9)	105 (32.3)
**ECOG PS**		
** 0**	328 (72.6)	236 (72.6)
** ≥1**	124 (27.4)	89 (27.4)
**Treatment at PD**		
** Everolimus + Exemestane**	50 (20)	31 (19.9)
** Endocrine therapy**	22 (8.8)	22 (14.1)
** Capecitabine**	97 (38.8)	54 (34.6)
** Taxanes**	50 (20)	42 (26.9)
** Anthracycline**	15 (6)	5 (3.2)
** Other therapies**	10 (2.5)	3 (1.9)
** Protocols**	6 (2.4)	5 (3.2)

MBC, metastatic breast cancer; CDK4/6i cyclin-dependent kinase 4/6 inhibitors; PS, performance status; PD, progressive disease.

154 (34.1%) patients had *de novo* metastatic disease and 298 (65.9%) had recurrent disease with a median disease-free interval of 72 months (range 3-375). 328 patients were postmenopausal at diagnosis, 121 were pre-perimenopausal and 3 patients were male. 219 (48.5%) patients had HER2 = 0 and 176 (38.9%) had HER2-low (e.g. immunohistochemical score of 1+ and 2+ without gene amplification) tumors assessed in primary tumors. 239 (52.9%) patients had visceral metastases and 117 (25.9%) had bone-only disease. 325 patients (71.9%) received ET + CDK4/6i as first-line line treatment, 85 and 42 as second- and third- line (mostly palbociclib). More than half of patients (54.7%) received palbociclib, 28.3% ribociclib and 17% abemaciclib. Palbociclib was the preferred CDK4/6i also among patients treated in first-line (43.1%) vs ribociclib (35.7%) and abemaciclib (21.2%), probably reflecting the timing of drug approvals. Aromatase inhibitors and fulvestrant were the endocrine agents in 53.1% and 46.9% of patients, respectively. Most patients had an ECOG PS = 0.

In the first-line cohort ribociclib was prescribed more frequently to patients with *de novo* as compared to those with recurrent disease (49.6 vs 28.10%) and to patients with endocrine-sensitive tumors (48% in endocrine-naïve vs 13.2% in primary endocrine resistant tumors), oppositely to abemaciclib which was more commonly prescribed to patients with endocrine-resistant tumors (39.5% in primary endocrine resistant vs 13% in endocrine naïve tumors, respectively). Regarding the association with endocrine partner, ribociclib was used almost exclusively combined with aromatase inhibitors differently from abemaciclib and palbociclib. No significant association between choice of CDK4/6i and disease status at diagnosis (bone-only vs visceral disease) was observed.

### Outcomes with CDK4/6i and ET

Outcomes with CDK4/6i and ET are summarized in Table S1 (see online supplementary material). Median follow-up was 43.6 months (1.5-88.3).

mPFS was 22.8 months overall and 29.7 months in patients treated in first-line line. PFS was significantly longer in *de novo* metastatic as compared with recurrent disease either overall (31.5 vs 18.8 months, *P* > .0001) and patients treated in first-line (34.4 vs 25.1 months, *P* = .004). Similarly, patients with bone-only disease had a significantly longer PFS as compared to those with visceral involvement (32.2 vs 16.1 months, *P* = .0002 overall and 34.4 vs 20.3 months, *P* = .0009 in patients treated in first-line). PFS was doubled with aromatase inhibitors vs fulvestrant, either overall and in patients treated upfront (33.3 vs 15.7 months, and 33.3 vs 17.5 months, respectively, *P* < .0001 in both cases). This finding could be biased since the majority of patients receiving fulvestrant had endocrine-resistant disease as compared to those receiving aromatase inhibitors (10.5% vs 91.8% of patients with endocrine-naïve and -sensitive tumors in the fulvestrant vs the aromatase inhibitors cohort, respectively).

In patients treated in first-line a statistically significant difference was observed across the three CDK4/6i. Head-to-head comparisons showed only a trend for statistically longer PFS favoring ribociclib vs palbociclib (*P* = .06), while OS did not differ either overall and in head-to-head comparisons with palbociclib obtaining a numerically longer OS (Table S1 and Figure S1—see online supplementary material).

No statistically significant difference in PFS was observed in patients with HER2 = 0 vs HER2-low tumors either overall (median 23.2 vs 20.9 months) and in patients treated in first-line (median 29.8 vs 29.7 months).

Overall survival (OS) results showed a statistically significant difference favouring patients with bone-only disease and non-endocrine resistant disease in both cohorts (Table S1—see online supplementary material). Patients with secondary endocrine-resistant tumors performed significantly better than those with primary endocrine-resistant tumors (*P* = .011 and *P* < .0001 overall and in patients treated in first-line, respectively). No difference among CDK4/6i either overall and in head-to-head comparisons was observed with palbociclib obtaining a numerically longer OS than abemaciclib and ribociclcib (Table S1—see online supplementary material). HER2 expression (0 vs low) was not associated with OS (data not shown).

Multivariable analysis of PFS showed a significant effect only for the line of therapy of CDK4/6i (later lines vs first line) (hazard ratio (HR) = 1.35 95% confidence intervals (CI) 1.08-1.68, *P* = .009), for *de novo* vs recurrent metastatic disease (HR= 0.62, 95% CI 0.41-0.923, *P* = .019) and for visceral vs bone-only disease at baseline (HR = 1.64, 95% CI 1.23-2.18, *P* < .001) (Table S2—see online supplementary material).

In multivariable analysis of OS only the presence of visceral vs bone-only metastases (HR = 2.38, 95% CI 1.60-3.51, *P* < .001) and of primary endocrine resistance (HR = 2.79, 95% CI 1.72-4.53, *P* < .001) vs no previous ET maintained a significant effect, while the line of CDK4/6i therapy had only a borderline effect (HR = 1.29, 95% CI 1.0-1.65, *P* = .050) (Table S2—see online supplementary material).

In patients treated in first-line significant prognostic factors of PFS included visceral vs bone-only disease (HR = 1.76, 95% CI 1.26-2.47, *P* = .001) and both primary (HR = 2.21, 95% CI1 0.41-3.47, *P* ≤ .001) and secondary endocrine resistance (HR = 2.26, 95% CI 1.56-3.27, *P* < .001) vs no prior ET. In multivariate analysis of OS only visceral metastases (HR = 1.70, 95% CI 1.08-2.67, *P* = .022) and primary endocrine resistance (HR = 3.04, 95% CI 1.76-5.24, *P* < .001) maintained a significant prognostic effect (Table S2—see online supplementary material).

### Outcomes after progression to ET and CDK4/6i

Table 2 summarizes results after progression to CDK4/6i and ET.

**Table 2.** Results after progression on CDK 4/6 inhibitors.

		Overall	*P*-Value	Patients treated in first-line	*P*-Value
		Median (95% CI)		Median (95% CI)	
***N* patients (% of total)**		250 (55.3%)		156 (48%)	
**Site of PD**					
** Not visceral**		79 (31.6%)		54/34.6%)	
** Visceral**		168 (67.2%)		101 (64.7%)	
** Missing**		3		1	
**PPFS (months)**		5.7 (5.1-6.9)		5.7 (5-6.9)	
**PPFS**	**Recurrent MBC**	6 (5.2.-7.2)		6.0 (5.1-7.4)	
	***De novo* MBC**	4.7 (3.9-6.9)	ns	5.4(3.9-7.6)	ns
**PPFS**	**Visceral baseline**	5.6 (4.8-7.2)		6.4 (5.3-7.5)	
	**Bone-only baseline**	5.3 (3.7-6.9)	ns	4.3 (2.8-5.7)	ns
**PPFS by site of PD**					
** not visceral**		5.7 (4.7-6.9)	ns	5.8 (4.3-8,.1)	ns
** visceral**		5.7 (4.7-7.4)		5.6 (4.5-7.4)	
**PPFS by CDK 4/6 i**					
** Abemaciclib**		6 (3.8-6.9)	ns	4.3 (2.8-6.7)	ns
** Palbociclib**		6 (5-7.6)		6.6 (4.8- 8.3)	
** Ribociclib**		5.5 (4.2-6.9)		5.5 (4.4-7.6)	
**PPFS by Endocrine partner**					
** Fulvestrant**		5.6 (4.8-7.4)	ns	5.6 (4.3-7.5)	ns
** Aromatase inhibitors**		5.7 (4.4-6.9)		5.8 (4.5-7.4)	
**PPOS after PD**		22.9(19.9-25.8)		24.3(18.7-28.7)	
**PPOS**	**Recurrent MBC**	21.4 (18.2-26.3)	ns	20.9 (16.9-28.7)	ns
	***De novo* MBC**	24.3 (18.7-28.6)		27.9 (18.7-33)	
**PPOS**	**Visceral baseline**	20.8 (17.1-24.5)		22.6 (16.4-28.6)	
	**Bone-only disease baseline**	32.9 (18.2-46.8)	.023	28 (16.9-33)	ns
**PPOS by CDK 4/6 i**					
** Abemaciclib**		28.6 (11.4-NE)		28.6 (11.4-NE)	
** Palbociclib**		23.2 (19-26.3)	ns	25.8 (15.2-32.9)	ns
** Ribociclib**		22.6 (18.2-30.4)		22.6 (18.2-30.4)	
**PPOS by ET**					
** Fulvestrant**		20.4 (16.9-24.6)	ns	18 (12.1-30.4)	ns
** Aromatase inhibitors**		27.9 (20.6-32)		27.9 (20.1-32.5)	
**PPOS by site of PD**					
** visceral**		20 (16.8-22.9)	<.001	18.7(13.8-28.3)	.007
** not visceral**		29.2 (25.4-41.7)		28 (24.3-NE))	
**PPFS by treatment at PD**					
** Everolimus + Exemestane**		5.4 (4.3-9)	<.001	6.7 (4.3-9.9)	0.049
** Endocrine therapy**		5.8 (3.4-8.3)		5.8 (3.4-8.3)	
** Capecitabine**		7.5 (5.5-8.5)		7 (4.7-8.5)	
** Taxanes**		4.3 (2.8-6)		4.2 (2.8-6.4)	
** Anthracycline**		4.5 (1.6-6.9)		5.4 (0.7-10.4)	
**PPOS by treatment after PD**					
** Everolimus + Exemestane**		33 (20.8-NE)	<.001	33 (20.8-NE)	<.001
** Endocrine therapy**		28.3 (20.1-NE)		28.3 (20.1-NE)	
** Capecitabine**		24.5 (19-28)		20.9 (12.8-34.5)	
** Taxanes**		13.9 (7.2-18)		9.2 (6.6-18.2)	
** Anthracycline**		18.7 (5.3-30.4)		18.7 (0.9-30-4)	

CDK4/6i, cyclin-dependent kinase 4/6 inhibitors; 95% CI, 95% confidence intervals; PD, progressive disease; PPFS, post progression-free survival; MBC, metastatic breast cancer; PPOS, post progression-free survival; NE, not estimated; NR, not reached.

Among 300 patients with progressive disease (PD) 250 patients (156 patients treated in first-line) received a treatment after PD. 40 patients were not deemed candidate for further systemic therapies, and no further information was available for 10 patients. PD was mostly visceral (168 overall and 101 in patients treated in first-line) vs non visceral (79 and 54, in the 2 groups, respectively).

The most common post-CDK4/6i treatment was capecitabine either alone or in combination with vinorelbine and cyclophosphamide in standard or metronomic schedule (38.8% and 34.6% overall and in patients treated in first-line, respectively), followed by taxanes (20% and 26.9% in the 2 cohorts), the combination of exemestane and everolimus (20% and 9.5%), endocrine single agent (mostly fulvestrant) (7.7% and 6.7%) and anthracycline-based regimen (6% and 1.5%) (Table 1). In addition, 10 patients received miscellaneous drugs (other cytotoxic agents, trastuzumab-deruxtecan and PARP inhibitors) and 6 patients were treated within experimental protocols.

Median PPFS (PFS2) with the first post-CDK4/6i therapy was 5.7 months (95% CI 5.1-6.9) overall and 5.7 months (95% CI 4.3-8.1) in patients receiving CDK4/6i in first-line. Post-progression treatment (excluding the 16 patients treated with miscellaneous and experimental drugs) was the only factor associated with longer PPFS (*P* < .001 overall and *P* < .05 in patients treated in first-line). Importantly, no difference was observed also in patients progressing at visceral sites vs not, while post-progression overall survival (PPOS) was significantly shorter in patients with visceral progression either overall and in patients receiving CDK4/6i upfront (median 20 vs 29.2 months *P* ≤ .001 and median 19.9 vs 28 months *P* < .02, respectively) (Table 2).

CDK4/6i treatment duration either as continuous and a categorized variable (≥ and < 12 months), was statistically significantly associated only with PPOS (HR= 0.98, 95% CI 0.97-1.00 *P* = .042). Median PPOS was 19.9 months (15-23.6) and 27.9 (20.4-31.6) in patients who had been treated with CDK4/6i <12 and ≥ 12 months (*P* = .018).

When all post-CDK4/6i treatments, excluding the 16 patients treated with miscellaneous and experimental drugs, were considered a significant difference in PPFS was observed either overall and in patients treated in first-line line (*P* < .001 and *P* < .05, respectively). PPOS too was statistically significantly different among treatments either overall and in the cohort of patients treated upfront (*P* < .001 for both comparisons) (Table 2, Figure S2—see online supplementary material for a color version of this figure).

### Outcomes according to first post-CDK4/6i treatments

In order to obtain information on the outcomes for different first post-CDK4/6i treatments we increased the size of each treatment group by combining patients receiving similar therapies namely exemestane and everolimus and ET (EET *n* = 72) and those receiving anthracyclines and/or taxanes (AT) (*n* = 65) to be compared with the group of patients treated with capecitabine-based regimens (C) (*n* = 97).

Treatments according to type of progression (visceral vs not visceral) and treatment duration with CDK4/6i and ET (<12 months vs ≥12 months) are reported in Figures S3 and S4 (see online supplementary material for a color version of these figures). Patients treated with AT were more likely to have experienced visceral progression and a CDK4/6i treatment duration < 12 months, oppositely to those in the EET group, while in the C group the two features were similarly distributed.

**Figure 1.**
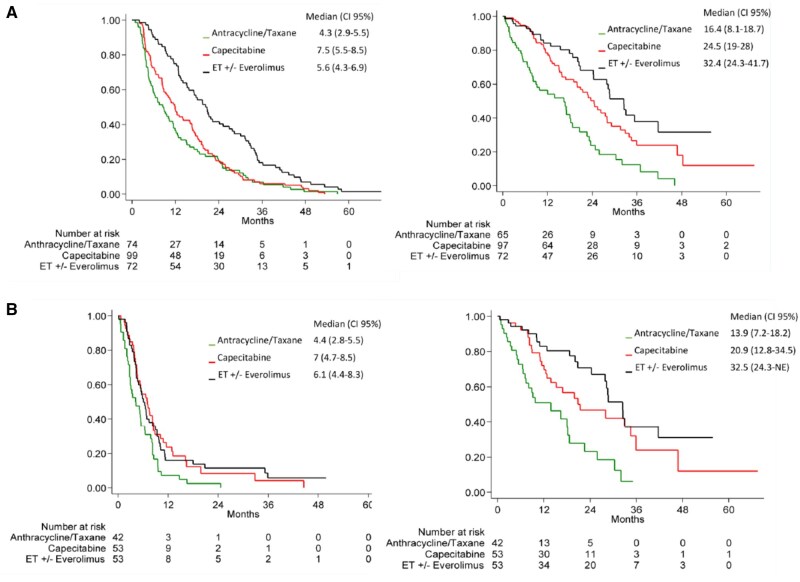
Post-progression free survival (PPFS) and post-progression overall survival (PPOS) according to combined treatment groups A) overall and B) in patients treated in first-line, ET: endocrine therapy.

mPPFS (mPFS2) was 5.6 months (95% CI 4.3-6.9) in the EET group, 7.5 months (95% CI 5.5-8.5) in the C group and 4.3 (95% CI 2.9-5.5) in the AT group. While no difference was observed between EET and C groups both showed a statistically significantly prolonged PPFS as compared to the AT group (*P* < .003 and *P* < .0001, respectively) (Table 3, Figure 1A).

**Table 3.** Comparisons of post progression-free survival and post progression-free overall survival according to treatment groups.

Variable	Overall	*P*-Value	Patients treated in first-line	*P*-Value
	Median (95% CI)		Median (95% CI)	
**PPFS by treatment**		<.001		.008
** Endocrine Therapy ±everolimus**	5.6 (4.3-6.9)	ns^a^	6.1 (4.4-8.3)	ns^a^
** Capecitabine regimens**	7.5 (5.5-8.5)	.003^b^	7.0 (4.7-8.5)	.009^b^
** Anthracyclines/taxanes**	4.3 (2.9-5.5)	<.001^c^	4.3 (2.8-5.5)	.007^c^
**PPFS by treatment visceral PD**		<.001		.016
** Endocrine therapy±everolimus**	5.4 (3.6-9)	ns^a^	6.1 (3.8-9.9)	ns^a^
** Capecitabine regimens**	7.6 (5.6-9)	.037^b^	7.2 (4.7-8.5)	ns^b^
** Anthracyclines/taxanes**	4.3 (2.8-5.5)	<.001	3.9 (2.6-5.6)	.007
**PPOS by treatment**		<.001		<.001
** Endocrine Therapy±everolimus**	32.5 (24.3-41.7)	ns^a^	32.5 (24.3-NE)	ns^a^
** Capecitabine regimens**	24.5 (19-28)	<.001^b^	20.9 (12.8-34.5)	<.001^b^
** Anthracyclines/taxanes**	16.4 (8.1-18.7))	.001^c^	13.9 (7.2-18.2)	.003^c^
**PPOS treatment visceral PD**		.001		.004
** Endocrine Therapy ±everolimus**	28.6 (18.7-NE)	ns^a^	28.6 (10.9-NE)	ns^a^
** Capecitabine regimens**	22.7 (15.9-26.3)	<.001^b^	20.9 (12.1-35.9)	.0057^b^
** Anthracyclines/taxanes**	16.4 (7.4-18.2)	.002^c^	9.2 (6.5-18.2)	.0078^c^

95% CI, 95%confidence intervals; PPFS, post progression-free survival; PD, progressive disease; PPOS, post progression overall survival; NE, not estimated.

a Endocrine therapy ± everolimus vs capecitabine regimens.

b Endocrine therapy± everolimus vs anthracycline/taxanes.

c Capecitabine regimens vs anthracycline/taxanes.

mPPOS was 32.5 months (95% CI 27.9-NE) in the EET and 24.5 months (95% CI 19-28) in the C group with a trend for a significant difference favoring the EET group(*P* = .064), while it was significantly shorter in the AT group (16.4 months 95% CI 8.1-18.7) as compared to the former groups (*P* < .001 for both comparisons) (Table 3 and Figure 1A).

Comparable results were observed in the cohort of patients treated in first-line (Table 3 and Figure 1B).

**Figure 2.**
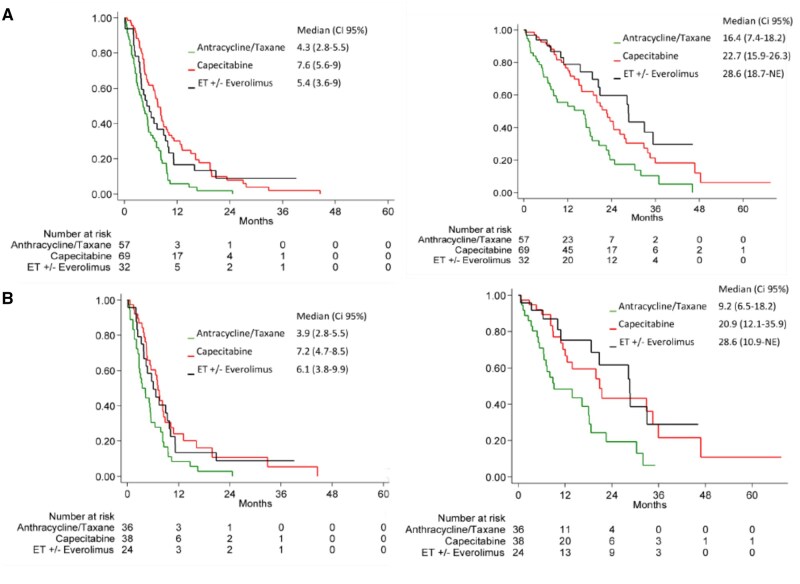
Post-progression free survival (PPFS) and post-progression overall survival (PPOS) according to combined treatment groups in patients progressing at visceral sites (A) overall and (B) in patients treated in first-line ET: endocrine therapy.

Since the choice of subsequent treatments was affected by the site of progression (Figure S3—see online supplementary material for a color version of this figure), we performed the same analyses separately in patients with visceral progression. mPPFS (mPFS2) was 5.4 months (95% CI 3.6-9) in the EET, 7.6 months (95% CI 5.6-9) in the C and 4.3 months (95% CI 2.8-5.5) in AT groups, respectively confirming the statistically significant inferiority of the latter group as compared with EET and C (*P* = .037 and *P* < .001, respectively) while no difference was observed between EET and C (Table 3 and Figure 2A).

mPPOS was 28.6 months (95% CI 18.7-NE) in the EET group and 22.7 (95% CI 15.9-2613) in the C group both statistically significantly longer than the 16.4 months (95% CI 7.4-18.2) of the AT group (*P* < .001 and *P* = .002, respectively) (Table 3 and Figure 2A).

In the cohort of patients with visceral progression treated in first-line only C resulted in a statistically significantly better PPFS than AT (*P* = .007), while a not significant trend favoured EET over AT(*P* = .058). No difference between EET and C was shown (Table 4 and Figure 2B). On the other hand, an improved PPOS for either EET and C over AT (*P* = .006 and *P* = .008, respectively) with no difference between the two former groups was shown (Table 3 and Figure 2B).

**Table 4.** Multivariate analysis for progression- free survival and overall survival at progression overall and in patients treated in first-line.

	PFS			OS		
Variable	Hazard ratio	95% CI	*P*-Value	Hazard ratio	95% CI	*P*-Value
**Therapy with capecitabine regimens** ^a^	0.52	0.37-0.73	0.001	0.53	0.35-0.79	<0.002
**Therapy with ET ± everolimus** ^a^	0.57	0.39-0.84	<0.005	0.37	0.23-0.62	<0.001
**PD visceral** ^b^	1.03	0.75-1.43	0.851	1.63	1.05-2.54	0.030

First-line						
	PFS			OS		
**Therapy with capecitabine regimens** ^a^	0.56	0.37-0.86	0.008	0.44	0.25-0.75	0.003
**Therapy with ET ± everolimus** ^a^	0.56	0.37-0.86	0.008	0.27	0.15-0.49	<0.001

PFS, progression-free survival; OS, overall survival; PD, progressive disease; 95% CI, 95% Confidence Interval; ET, endocrine therapy; AT, anthracycline taxane.

a vs AT.

b vs not visceral PD.

CDK4/6i treatment duration did not significantly differ in the 3 post-CDK4/6i treatment groups (EET = 23.6 months, 95% CI 20-27; *C* = 15.1 months, 95% CI 12.7-17.1; AT= 13 months, 95% CI 10.1-15.9, *P* = 0.53).

Results of multivariable analysis of PPFS and PPOS are reported in Table 4. Only post-progression treatment showed a significant effect on all outcomes, while the occurrence of visceral progression maintained a significant independent prognostic effect only on overall PPOS. CDK4/6i treatment duration was not significantly associated with both outcomes regardless it was considered as a continuous or a categorized variable. In the group of patients who had been on treatment with CDK4/6i <12 months EET and C still performed statistically significantly better than AT for both PPFS (median 6.8, 7.3 and 3.8 months, respectively, *P* < .001) and PPOS (median 42, 23.7 and 12 months, respectively, *P* < .001).

### Outcomes after progression to the first post-CDK 4/6i treatment

A total of 179 patients experienced a progression after the first post-CDK4/6i treatment and 172 received a subsequent systemic treatment. Chemotherapy was the preferred treatment: AT in 36.6% of patients followed by C regimens 33.1% while 8.7% of patients received endocrine± targeted therapies. Remaining patients (about 21%) received miscellaneous treatments.

Median post progression free-survival 2 (mPPFS2) was 5.2 months (95% CI 4.4-5.7). No difference among the 3 groups of treatment was observed either overall and in patients treated upfront: 5.8 months (95% CI 4.1-8,1) and 5.8 months (95% CI 4.1-11.2) for C; 4.4 months (95% CI 2.4-8.3) and 4.1 (95% CI 1.7-8.3) for EET and 5.6 months (95% CI 4.4-6.4) and 5.7 months (95% CI 2.9-6.4) for AT groups.

Median post progression overall survival 2 (mPPOS2) was 16.1 months (95% CI 13.7-19.8), 21 months (95% CI 15.4-30) for C, Not Reached for EET (95% CI 5.3-NE) and 15.9 (95% CI 8.9-21.9) for AT with no significant difference among groups (Figure S5—see online supplementary material for a color version of this figure). However, after excluding the 15 patients treated with EET, C achieved a significantly longer PPOS2 compared to AT both overall and in patients treated in first-line (*P* = 0.037 and *P* = 0.018).

## Discussion

Despite the increasing knowledge of molecular pathways involved in endocrine resistance has made available new endocrine-based targeted therapies as oral SERDs and PI3KCA/AKT/mTOR inhibitors,^12–17^ which may be useful for more than one half of the patients progressing to first line treatment,^21^ the issue of the optimal treatment after progression remains crucial for a substantial proportion of patients who do not harbor targetable mutations or do not have access to molecular analyses.

In this real-world study we retrospectively analyzed a large cohort of consecutive patients with HR+/HER2− ABC more than two-thirds of whom receiving a first-line therapy, treated in routine practice in two large-volume research hospitals. Patient characteristics were similar to those of pivotal trials: about one-third were metastatic *de novo.* about 50% and 25% had visceral and bone-only disease, respectively. Our OS results with abemaciclib and ribociclib plus ET are slightly different from those of pivotal trials but the smaller size of the 2 groups may account for these differences. Aware of the limitations of non-randomized comparisons, we observed a not statistically significant trend for worse PFS but a numerically longer OS with palbociclib in patients treated in first-line.

As expected, treatment choice following progression was affected by the site of progression with most patients receiving taxane- and anthracycline-based chemotherapy after visceral progression (87.6%), whereas patients with bone progression received mostly endocrine-based therapy or capecitabine. Despite this expected imbalance in treatment choice, ET ± everolimus resulted in better outcomes either overall and in patients with visceral progression. Moreover, the observed trend for improved OS for endocrine therapies over capecitabine, suggests the persistence of endocrine-sensitivity beyond failure of CDK4/6i and, importantly, that delaying the administration of chemotherapy rather than being detrimental allows the availability of a further line of treatment at the onset of endocrine resistance. The effectiveness of capecitabine-based regimens as salvage chemotherapy after occurrence of endocrine resistance is also supported by the improved PPOS2 after the second progression in patients treated in first-line.

Our results also confirm the role of capecitabine-based regimens as preferable chemotherapy option after CDK/6i failure formerly proposed by the METEORA-II trial which showed an improved PFS and time-to-treatment failure for the VEX regimen (metronomic capecitabine, vinorelbine and cyclophosphamide) as compared with paclitaxel.^22^

As expected, duration of benefit with CDK4/6i as a proxy for endocrine sensitiveness, was associated with treatment decision since patients progressing within 12 months were more likely to receive chemotherapy, but unexpectedly it was associated with post-progression outcomes only in univariate analysis of OS and more importantly, it lost its effect in multivariate analysis when considering treatment and visceral progression. In addition, even among patients progressing after <12 months, endocrine ± targeted therapies and capecitabine performed better than anthracyclines and/or taxanes.

In our study, both single agent ET and the combination with everolimus in a HR+/HER2− population unselected for driver mutations as *ESR1*, *PI3KCA*/*AKT* favourably compared with results obtained with targeted agents in cohorts selected for specific mutations [12-16]. Moreover, single agent ET (mostly fulvestrant) performed better than reported in control arms of RCTs. Despite being a real-world series our population mirrors those patients treated within second line RCTs with about 75% of patients having received only one prior ET and < 15% of patients pretreated with chemotherapy for advanced disease [12-16]. On the other hand, we cannot rule out that more stringent intervals in disease restaging reported in clinical studies than routine clinical practice can explain the different results of PFS although OS should not be affected by this bias. In addition, a proportion of patients might have carried unknown mutations of the *PI3KCA/mTOR* pathway and may have benefited of a specific treatment as everolimus.

A number of real-world studies with variable sample size have reported outcomes with post-CDK4/6i treatments but comparisons of the effectiveness of the different therapies were not always included.

The largest report derived from the Flatiron Health database, a longitudinal real-world registry comprising de-identified structured and unstructured data from 280 sites across the United States. This analysis included 1210 patients, of whom 839 received a documented second-line therapy after progression on CDK4/6i plus ET in first line.^23^ Differently from our study about 36% of patients continued a CDK4/6i, mostly switching only the endocrine partner, while chemotherapy was, administered to 29.7% of patients and endocrine therapy (mostly fulvestrant) was prescribed in 12.4% and everolimus in 11.7%. A marginal proportion of patients received other therapies (2.4%) or were enrolled in clinical trials (about 6%). The better outcomes were obtained maintaining CDK4/6i therapy (PFS= 8.25 months and OS= 35.7 months) while a similar rwPFS for chemotherapy, fulvestrant and everolimus was observed (3.71, 3.25 and 3.32 months, respectively). OS favoured everolimus but not fulvestrant monotherapy as compared to chemotherapy.^23^ Differently from our study and due to the inherent limitations of data collection type, no information about outcomes according to the site of progression or other clinical and pathological characteristics were available.

The cooperative GIM (Gruppo Italiano Mammella) reported the results of the retrospective/prospective GIM14 study evaluating treatment patterns and outcomes in patients with metastatic breast cancer treated at 26 Italian centres.^24^ Among 701 patients receiving CDK4/6i in first-line 239 received treatment after progression, 45.5% of whom ET ± everolimus, and 40% capecitabine and taxane-based regimens. Consistently with our series patients with shorter CDK4/6i treatment duration received more frequently chemotherapy. No difference in PPFS (PFS2) was observed among treatments. No data on outcomes according to site of progression was reported.^24^

A systematic review including 18 studies of post-CDK 4/6i treatment reported median real-world PFS of 3.9 months (3.3-6.0 months) for single-agent ET, 3.6 months (2.5-4.9 months) for mTORinhibitors ± ET, 3.7 months for a mix of ET and/or mTORinhibitors (3.0-4.0 months), and 6.1 months (3.7-9.7 months) for chemotherapy. Median real-world OS was not calculated since only 3 studies reported this outcome. No information on treatment after the second progression was reported as well.^25^

Our study shares the inherent limitations of real-world studies. Firstly, the lack of standardized timing and methods of disease evaluation might affect the calculation of outcome measures. Another limitation is represented by the increasingly smaller sample size for each treatment group along subsequent treatment lines, which might weaken statistical significance of post-progression outcome comparisons among these groups. On the other hand, key strength of our study is the inclusion of only two large-volume centers which reduces the variability in patient management. To our knowledge this it is the first real-world post-CDK4/6i study which reports the impact of site of progression on all the second-line treatment outcomes and shows that PPFS and PPOS were not affected by factors associated with CDK4/6i failure. Finally, we report for the first time outcomes after the progression after the first post-CDK4/6i treatment.

In conclusion, our results show in a large real-world series that most patients with HR+/HER2− ABC failing therapy with CDK4/6i and ET who were not selected for the occurrence of molecular mutations retain endocrine sensitivity and may derive a meaningful clinical benefit from a subsequent endocrine manipulation ± a targeted therapy delaying the need for chemotherapy regardless of site of progression and duration of prior CDK4/6i therapy. Our findings support a beneficial role for extending endocrine therapies in routine clinical practice when actionable mutations are not detected or not evaluable.

## Supplementary Material

oyag003_Supplementary_Data

## Data Availability

The datasets generated and analyzed during the current study are available on the repository Zenodo: https://zenodo.org/records/
