# Supplementary material for: Real-world patterns of post-progression treatment and outcomes in patients with HR+/HER2− advanced breast cancer treated with CDK4/6 inhibitors
Source: Oncologist. 2026 Jan 11;31(3):oyag003. doi: 10.1093/oncolo/oyag003 (PMC12923151; doi:10.1093/oncolo/oyag003)
Supplement: oyag003_Supplementary_Data [file oyag003_supplementary_data.zip › Supplementary Tables.docx]

**Supplementary Table 1. Results with CDK4/6 inhibitors**

|  | OVERALL  Median (95%CI) | p-value | Patients treated in first- line  Median (95%CI) | p-value |
| --- | --- | --- | --- | --- |
| N patients | 452 |  | 325 |  |
| PFS (months) | 22.6 (19.5-26) |  | 29.7 (24-32.2) |  |
| PFS Recurrent MBC  *De novo* MBC | 18.8 (16.7-23.2)  31.5 (23.7-41.5) | <.001 | 25.1 (19.2-30.7)  34.4 (28.3-47.4) | .004 |
| PFS Visceral disease  Bone-only disease | 16 (12.6-19.5)  32.2 (26.5-35.2) | <.001 | 20.3 (14.2-24.2)  34.1. (30.6-42.5) | <0.001 |
| PFS by Endocrine resistance  Naïve  Hormone sensitive  Primary resistance  Secondary resistance | 34.4 (28.3-46.8)  33.3 (27.7-46.6)  9.3 (4.6-16.8)  17.1 (12.4-19.6) | <.001 | 34.4 (28.3-44.9)  34.7 (30.6-46.8)  12.1 (5.5-23.2)  17.2 (11.8-24.2) | <.001 |
| PFS by CDK 4/6 i  Abemaciclib  Palbociclib  Ribociclib | 30.8 (17.2-50.4)  18.1 (13.8-20.8)  32.2 (26.5-43.4) | <.001 | 30.8 (16-50.4)  25.1 (18.3-31.5)  31.5 (26.5-42.5) | 0.048  *ns  **ns  ***ns |
| PFS by endocrine partner  Fulvestrant  Aromatase inhibitors | 15.7 (11.9-18.1)  33.3 (29.8-42.5) | <.001 | 17.5 (12.2-24.2)  33.3 (30.-42.5) | <.001 |
| OS | 51.2 (47.2-58.1) |  | 56.6 (51.2-65.4) |  |
| OS Recurrent MBC  *De novo* MBC | 48.4 (40.5-58.7)  56.6 (50-65.4) | NS | 54.4 (48.4-NE)  57.4 (51.2-NE) | NS |
|  |  |  |  | .01 |
| OS Visceral disease  Bone-only disease | 39.3 (36.1-47.3)  65.4 (53-NE) | <.001 | 50.4 (41.1-60.3)  56.6 (50.4-NE) |  |
| OS by Endocrine resistance  Naïve  Hormone sensitive  Primary resistance  Secondary resistance | 57.4 (51.5-NE)  NR (50.4-NR)  26.3 (18-38.5)  43.6 (36.1-50.8) | <.001  .011^ | 56.6 (51.2- NE)  NR (50.4-NE)  28.4 (18-38.5)  48.4 (35.7-NE)^ | <.001  <.001^ |
| OS by CDK 4/6 i  Abemaciclib  Palbociclib  Ribociclib | 51.5 (41.9-NE)  45.6 (38.1-53)  56.2 (50.4-NE) | .015 | 51.5 (41.9-NE)  58.7 (45.5-NE)  54.4 (50-60.6) | ns  *ns  **ns  ***ns |
| OS by endocrine partner  Fulvestrant  Aromatase inhibitors | 39.3 (35.7-47.3)  60.3 (53-NE) | <.001 | 45.5(35.7-NE)  58.7 (52-NE) | .0022 |

CDK4/6i cyclin-dependent kinase 4/ 6 inhibitors PFS progression-free survival; 95%CI 95% confidence intervals ; MBC metastatic breast cancer; NE not estimated; NR not reached

* abemaciclib vs palbociclib **abemaciclib vs ribociclib ***palbociclib vs ribociclib

^ secondary vs primary resistance

**Supplementary Table 2. Multivariate analysis for progression-free survival and overall survival at progression after CDK4/6 inhibitors overall and in patients treated in first-line**

| **PFS** | | | |  | **OS** | | |
| --- | --- | --- | --- | --- | --- | --- | --- |
| **Variable** | **Hazard ratio** | **95% CI** | **P-value** |  | **Hazard ratio** | **95%CI** | **p-value** |
| **CDK 4/6i later line of therapy *** | 1.35 | 1.08-1.68 | 0.009 |  | 1.29 | 1.00-1.65 | 0.050 |
| **Endocrine resistance primary **** | 1.58 | 0.94-2.67 | 0.086 |  | 2.79 | 1.72-4.52 | <0.001 |
| **Endocrine resistance secondary **** | 1.20 | 0.76-1.90 | 0.43 |  | 1.36 | 0.88-2.11 | 0.167 |
| **Endocrine sensitive**** | 0.72 | 0.44-1.18 | 0.191 |  | 0.88 | 0.54-1.41 | 0.588 |
| **Visceral disease ^** | 1.64 | 1.23-2.18 | <0.001 |  | 2.38 | 1.60-3.54 | <0.001 |
| **Soft-tissue disease ^** | 1.14 | 0.79-1.64 | 0.488 |  | 1.14 | 0.68-1.93 | 0.621 |
| ***De novo* MBC #** | 0.62 | 0.41-0.92 | 0.019 |  | / | / | / |
| **First-line** | | | | | | | |
|  | **PFS** | | |  | **OS** | | |
| **Endocrine resistance primary **** | 2.21 | 1.41-3.47 | <0.001 |  | 3.04 | 1.76-5.24 | <0.001 |
| **Endocrine resistance secondary **** | 2.26 | 1.56-3.27 | <0.001 |  | 1.45 | 0.87-2.42 | 0.151 |
| **Endocrine sensitive**** | 1.04 | 0.71-1.50 | 0.854 |  | 0.80 | 0.48-1.35 | 0.405 |
| **Visceral disease ^** | 1.76 | 1.26-2.47 | 0.001 |  | 1.70 | 1.08-2.67 | 0.022 |
| **Soft-tissue disease ^** | 1.18 | 0.77-1.81 | 0.447 |  | 0.89 | 0.48-1.65 | 0.720 |

PFS progression-free survival OS overall survival PD progressive disease 95%CI 95% Confidence Interval

CDK4/6i CDK4/6i cyclin-dependent kinase 4/ 6 inhibitors MBC metastatic breast cancer

*vs 1^st^ line; ** vs endocrine-naïve; ^ vs bone-only disease; # vs recurrent MBC
