# Supplementary material for: Real-world patterns of post-progression treatment and outcomes in patients with HR+/HER2− advanced breast cancer treated with CDK4/6 inhibitors
Source: Oncologist. 2026 Jan 11;31(3):oyag003. doi: 10.1093/oncolo/oyag003 (PMC12923151; doi:10.1093/oncolo/oyag003)
Supplement: oyag003_Supplementary_Data [file oyag003_supplementary_data.zip › Supplementary Figures The Oncologist 2 revision..docx]

Supplementary Figure 1: Progression-free survival and overall survival according to CDK4/6 inhibitor in patients treated in first-line.

 Supplementary Figure 2 Post-progression free survival (A) and post-progression overall survival (B) according to post-progression treatments in patients treated in first-line.ET= endocrine therapy

 Supplementary Figure3: Post progression treatments according to site of progression : EET: ET +/- Everolimus, C: Capecitabine backbone ,AT: Anthracycline and/or Taxane


Supplementary Figure 4: Post progression treatments according to CDK4/6i treatment duration . EET: ET +/- Everolimus, C: Capecitabine backbone ,AT: Anthracycline and/or Taxane

Supplementary Figure 5 Post-progression overall survival after the second progression (PPOS2) according to treatment groups in patients treated in first-line
